# Supplementary material for: Proteomic markers of colorectal cancer risk: results from the European Prospective Investigation into Cancer and Nutrition
Source: medRxiv. 2026 Oct 7:2026.10.05.26364761. Preprint. [Version 1] doi: 10.64898/2026.10.05.26364761 (PMC13692558; doi:10.64898/2026.10.05.26364761)
Supplement: Supplementary file 1 [file NIHPP2026.10.05.26364761v1-supplement-1.pdf]

medRxiv preprint doi: https://doi.org/10.64898/2026.10.05.26364761; this version posted October 7, 2026. The copyright holder for this preprint (which was not certified by peer review) is the author/funder, who has granted medRxiv a license to display the preprint in perpetuity.
It is made available under a CC-BY 4.0 International license .

7. Legends

Supplementary Figure 1 Comparison of models and follow-up exclusions for proteins associated with colorectal, colon, and rectal cancer risk in the main analysis. Forest plots show the hazard ratio and 95% confidence interval for each protein reaching the false discovery rate threshold (FDR-adjusted p < 0.05) in the sex-combined analysis of colorectal (36 proteins), colon (50 proteins), or rectal (2 proteins) cancer. Proteins are grouped by cancer and labelled as cancer: protein. (Left) Comparison of the minimally-adjusted (model 1) and fully-adjusted (model 2) models, with no follow-up exclusion. (Right) Comparison of the fully-adjusted model (model 2) with no exclusion and after excluding the first 2- and 5-years of follow-up. Filled points indicate FDR-adjusted p < 0.05 in the analysis shown.

Supplementary Figure 2 Correlation heatmaps for proteins associated with cancer risk within each cancer-sex analysis. Supplementary Figure 2 is a multi-page PDF with one page for each cancer (colorectal, colon, rectum, proximal colon, distal colon) and sex (combined, female, male) combination. Each page shows 3 heatmaps of the pairwise Pearson correlations between the proteins reaching the false discovery rate threshold (FDR-adjusted p < 0.05) in that analysis, calculated among all participants, among cases only, and among non-cases only, using pairwise complete observations. Correlations are reported in Supplementary Table 3.

Supplementary Figure 3 Correlation networks for proteins associated with cancer risk within each cancer-sex analysis. Supplementary Figure 3 is a multi-page PDF with one page for each cancer (colorectal, colon, rectum, proximal colon, distal colon) and sex (combined, female, male) combination. Each page shows 3 network maps, calculated among all participants, among cases only, and among non-cases only, in which proteins are connected where the pairwise Pearson correlation exceeds 0.4; edge width and shading are scaled to the strength of the correlation.

Supplementary Figure 4 Comparison of models and follow-up exclusions for proteins associated with cancer risk within each cancer-sex analysis. Supplementary Figure 4 is a multi-page PDF with one page for each cancer (colorectal, colon, rectum, proximal colon, distal colon) and sex (combined, female, male) combination. Each page shows 2 forest plots for the proteins reaching the false discovery rate threshold (FDR-adjusted p < 0.05) in that analysis: (left) a comparison of the fully-adjusted model (model 2) with no exclusion and after excluding the first 2- and 5-years of follow-up, and (right) a comparison of the minimally-adjusted (model 1) and fully-adjusted (model 2) models with no follow-up exclusion. Filled points indicate FDR-adjusted p < 0.05. Combinations with no proteins reaching the threshold are shown as an empty page.

Supplementary Figure 5 Cox proportional hazard models of the association between 7,289 aptamers, targeting 6,402 unique proteins, and all cancer-sex combinations. The volcano plot shows the hazard ratio and -log10 p-value for each aptamer. Highlighted (orange) points reached the Benjamini-Hochberg false discovery rate p-value threshold (5%) as indicated by the horizontal line. Labelled points are proteins associated (FDR) in ≥ 3 cancer-sex analyses.

Supplementary Figure 6 Evidence of heterogeneity by sex and/or by site in Cox proportional hazard models of colorectal cancer. All panels show the results from the respective sex-cancer analysis and orange points in all panels highlight the 36 proteins associated in the sex-combined colorectal cancer analysis. Purple points highlight proteins where there was evidence of heterogeneity by sex; green points highlight proteins where there was evidence of heterogeneity by site; blue points highlight proteins where there was evidence of heterogeneity by sex and site; the horizontal line (orange) indicates the false discovery rate (FDR) p-value threshold for the respective analysis.

Supplementary Figure 7 Comparison of colorectal cancer associated proteins in EPIC with results from MR analyses using different genome-wide summary-level datasets to identify protein instruments. Scatter plots show the hazard ratio (EPIC) and odds ratios (MR) for proteins associated with colorectal, colon, rectal, proximal colon, and distal colon cancer, with 1 panel per protein-QTL dataset (Ferkingstad et al., 2021; Pietzner et al., 2021; Zhang et al., 2022). Solid points indicate a nominal p-value threshold (0.05) was reached in the MR analysis.

Supplementary Table 1 Baseline characteristics by cancer site and sex group. Baseline characteristics are shown for each cancer site and sex-specific analysis group. Continuous variables are reported as median (SD); categorical variables are reported as n (%), among cases unless otherwise indicated.

Supplementary Table 2 Proteins associated with cancer site and sex groups at FDR < 0.05 in the primary EPIC analysis. Columns: protein, SomaLogic protein name; UNIPROT, UniProt accession; cancer, cancer outcome; sex, analysis sex group; HR, hazard ratio; CI_lower/CI_upper, 95% confidence interval; pval, nominal P value; pval_FDR, FDR-adjusted P value; het_sex, sex-heterogeneity P value where applicable; het_site, anatomical-site heterogeneity P value where applicable.

Supplementary Table 3 Pairwise Pearson correlations between proteins associated with cancer risk within each cancer-sex analysis. For each cancer and sex combination in which at least 1 protein reached the false discovery rate threshold (FDR-adjusted p < 0.05), correlations were calculated between all pairs of those proteins using pairwise complete observations, separately among all participants, among cases only, and among non-cases only. Thirteen of the 15 cancer-sex combinations are represented; proximal colon cancer in men and rectal cancer in women are absent because no protein reached the threshold in those analyses. Columns: cancer, cancer outcome; sex, analysis sex group; subset, whether the

medRxiv preprint doi: https://doi.org/10.64898/2026.10.05.26364761; this version posted October 7, 2026. The copyright holder for this preprint (which was not certified by peer review) is the author/funder, who has granted medRxiv a license to display the preprint in perpetuity.
It is made available under a CC-BY 4.0 International license .

correlation was calculated among all participants, cases only, or non-cases only; n_samples, number of participants contributing to the correlation; n_proteins, number of proteins included in that analysis; protein_1/protein_2, the protein pair; correlation, Pearson correlation coefficient. Correlations are shown graphically in Supplementary Figure 2, and as networks for pairs with a correlation exceeding 0.4 in Supplementary Figure 3.

Supplementary Table 4 All Cox proportional hazards results in EPIC across models and follow-up exclusions. Results are reported for all 7,289 aptamers, targeting 6,402 unique proteins, in each of the 15 cancer-sex analyses (colorectal, colon, rectum, proximal colon, and distal colon, each for combined sexes, women, and men), under the minimally-adjusted (model 1) and fully-adjusted (model 2) models, and with no follow-up exclusion and after excluding the first 2- and 5-years of follow-up. Unlike Supplementary Table 2, this table is not restricted to proteins reaching the false discovery rate threshold. Columns: protein, SomaLogic protein name; UNIPROT, UniProt accession; cancer, cancer outcome; sex, analysis sex group; model, 1 for the minimally-adjusted and 2 for the fully-adjusted model; followup, number of years of follow-up excluded (0 indicates no exclusion); HR, hazard ratio; CI_lower/CI_upper, 95% confidence interval; pval, nominal P value; pval_FDR, Benjamini-Hochberg FDR-adjusted P value.

Supplementary Table 5 Comparison of protein-cancer associations across EPIC, ARIC, and UK Biobank for all FDR-significant proteins within EPIC. Only proteins available in at least 1 comparison cohort are listed. Where ARIC measured a protein with an additional aptamer that did not match the aptamer used in EPIC, that row is labelled protein (SeqId) to distinguish it from the matched measure.

Supplementary Table 6 Mendelian randomisation estimates for protein effects on cancer outcomes across external protein-QTL studies and MR methods for all FDR-significant proteins within EPIC. Columns: protein, SomaLogic protein name; study, the author, year, and PubMed ID of the protein-QTL study used to identify instruments; UNIPROT, UniProt accession; cancer, cancer outcome; sex, analysis sex group; method, MR method; OR, odds ratio; CI_lower/CI_upper, 95% confidence interval; pval, nominal P value; nsnp, number of variants in the instrument; n_exposure/n_outcome, sample sizes of the exposure and outcome genome-wide association studies.

Supplementary Table 7 Colocalisation posterior probabilities for protein-cancer genetic association signals across external protein-QTL studies for all FDR-significant proteins within EPIC. Posterior probabilities are reported for each hypothesis (h0 to h4), where h4 is the posterior probability of a shared causal variant, together with the prior probabilities used.

Supplementary Table 8 Comparison of cancer associated proteins in EPIC with results from ARIC, UKB, and MR analyses. Scatter plots show the hazard ratios (EPIC, ARIC, and UKB) and odds ratios (MR) for proteins associated with colorectal, colon, rectal, proximal colon, and distal colon cancer in the main analysis performed in the European Prospective Investigation into Cancer and Nutrition (EPIC) that were tested in the Atherosclerosis Risk in Communities (ARIC) study, the UK Biobank (UKB), and in Mendelian randomisation (MR) analyses using genome-wide summary-level data. Solid points indicate a nominal p-value threshold (0.05) was reached in the non-EPIC analysis. In the EPIC-ARIC and EPIC-UKB panels only proteins with consistent directions of effect in the 2 studies are labelled; in the EPIC-MR panel all points are labelled. NB: Tetranectin (EPIC, ARIC, and MR) and CLEC3B (UKB) are the same protein.

Supplementary Table 9 Protein annotation information for FDR-significant proteins. This table lists unique proteins identified in Supplementary Table 2 and provides functional annotation from UniProtKB. Cancer-sex combinations indicate all analyses in which each protein was FDR-significant; UNIPROT: UniProt accession identifier; cancer-sex: Cancer and sex analysis combinations where the protein was identified as FDR-significant; function_summary: UniProt functional description of the protein; tissue_specificity: UniProt tissue-specificity annotation, where available; disease_involvement: UniProt disease association annotation, where available; go_biological_process: Gene Ontology biological process terms; go_molecular_function: Gene Ontology molecular function terms; go_cellular_component: Gene Ontology cellular component terms.

Supplementary Table 10 Broad functional categories of colon cancer associated proteins. This table summarises the proteins identified in the colon cancer, sex-combined analysis by broad functional category. Categories were assigned using the functional, tissue, disease, and gene ontology annotations reported in Supplementary Table 9. Category: broad biological category assigned to the proteins; summary: brief description of the shared biological features or annotation themes supporting the category.
